# Genetic variants in PI3K/AKT pathway are associated with severe radiation pneumonitis in lung cancer patients treated with radiation therapy

**DOI:** 10.1002/cam4.564

**Published:** 2015-12-08

**Authors:** Yang Tang, Bo Liu, Jing Li, Huanlei Wu, Ju Yang, Xiao Zhou, Mingxiao Yi, Qianxia Li, Shiying Yu, Xianglin Yuan

**Affiliations:** ^1^ Department of OncologyTongji HospitalHuazhong University of Science and TechnologyWuhanHubei ProvinceChina

**Keywords:** AKT, lung cancer, PI3CA, PI3K, radiation pneumonitis, SNP

## Abstract

PI3K/AKT pathway plays important roles in inflammatory and fibrotic diseases while its connection to radiation pneumonitis (RP) is unclear. In this study, we explored the associations of genetic variants in PI3K/AKT pathway with RP in lung cancer patients with radiotherapy. Two hundred and sixty one lung cancer patients with radiotherapy were included in this prospective study (NCT02490319) and genotyped by MassArray and Sanger Sequence methods. By multivariate Cox hazard analysis and multiple testing, GA/GG genotype of *AKT2*: rs33933140 (HR = 0.272, 95% CI: 0.140–0.530, *P *=* *1.3E–4, *P*_*c*_ = 9.1E–4), and the GT/GG genotype of *PI3CA*: rs9838117 (HR = 0.132, 95% CI: 0.042–0.416, *P *=* *0.001, *P*_*c*_ = 0.006) were found to be strongly associated with a decreased occurrence of RP ≥ grade 3. And patients with the CT/TT genotype of *AKT2*: rs11880261 had a notably higher incidence of RP ≥ grade 3 (HR = 2.950, 95% CI: 1.380–6.305, *P *=* *0.005, *P*_*c*_ = 0.025). We concluded that the genetic variants of PI3K/AKT pathway were significantly related to RP of grade ≥ 3 and may thus be predictors of severe RP before radiotherapy, if further validated in larger population.

## Introduction

Lung cancer is the leading cause of cancer‐related mortality worldwide, with approximately 1.59 million deaths globally each year 1. The American Cancer Society estimated that lung cancer accounts for 159,260 deaths annually, which is approximately 28% of all male cancer deaths and 26% of all female cancer deaths in the United States 2. For lung cancer patients, radiotherapy (RT) with or without chemotherapy remains the cornerstone treatment.

As the most common complication and the major dose‐limiting toxicity associated with radiotherapy, radiation pneumonitis (RP) is a type of inflammation and subsequent fibrosis that occurs after irradiation, and might hinder the tumor‐controlling effects of radiotherapy by limiting the radiation dose that can be applied and the size of the irradiated volume 3, 4. Further, RP can cause poor quality of life or life‐threatening symptoms in 5–50% of all patients who are irradiated for lung cancer 5, 6. Consequently, establishing reliable predictors for the occurrence of RP is of great significance such that the therapeutic effects of RT can be maximized while minimizing its adverse effects. In addition to the patient‐ and treatment‐related factors reported by previous studies, including Karnofsky performance status (KPS), chronic lung disease, smoking status, chemotherapy, dosimetric parameters, and plasma values of TGF*β* 5, 7, 8, 9, 10, 11, some genetic variants were recently found to be associated with the occurrence and development of RP 12, 13. Single‐nucleotide polymorphisms (SNPs) of *TGFβ* were found by us to be related to RP previously 14. However, the roles of genetic variants in downstream pathway of TGF*β* in RP remain unclear, which deserves our further investigation.

As an important downstream of TGF*β*, PI3K/AKT pathway was demonstrated to participate in the pathogenesis of inflammation and fibrosis diseases. There is now strong evidence that PI3K/AKT can regulate several determinant events in the inflammatory response to injury 15. Several studies demonstrated that PI3K inhibitors or genetic loss of PI3K cause reduction in recruitment and activation of lymphocytes, neutrophils, macrophages, and eosinophils in a variety of in vitro and in vivo researches 16, 17. AKT can also regulate development and differentiation of lymphocytes by FOXO 18. Meanwhile, PI3K/AKT pathway also plays important roles in pulmonary fibrosis 19, 20. In the pathogenesis of RP, lung epithelia cells are demonstrated to transdifferentiate into fibroblast‐like cells by epithelial‐to‐menschymal transition (EMT) 4. PI3K/AKT signaling pathway is a critical mediator of EMT 21. Moreover, PI3K/AKT can suppress the apoptosis of fibroblasts and affect adhesion of fibroblasts with fibronectin and collagen by interaction with other signaling pathways 19, 22. However, to the best of our knowledge, no studies have addressed the role of genetic variants of PI3K/AKT signaling pathway in RP. So we supposed that SNPs in PI3K/AKT were associated with the occurrence and development of RP. To verify this hypothesis, we selected seven potentially functional SNPs from three genes in PI3K/AKT signaling pathway, *PI3CA*,* AKT1,* and *AKT2* to discover their potential associations with the occurrence of severe RP in lung cancer patients treated with radiation therapy.

## Materials and Methods

### Patient population

**Figure 1:**
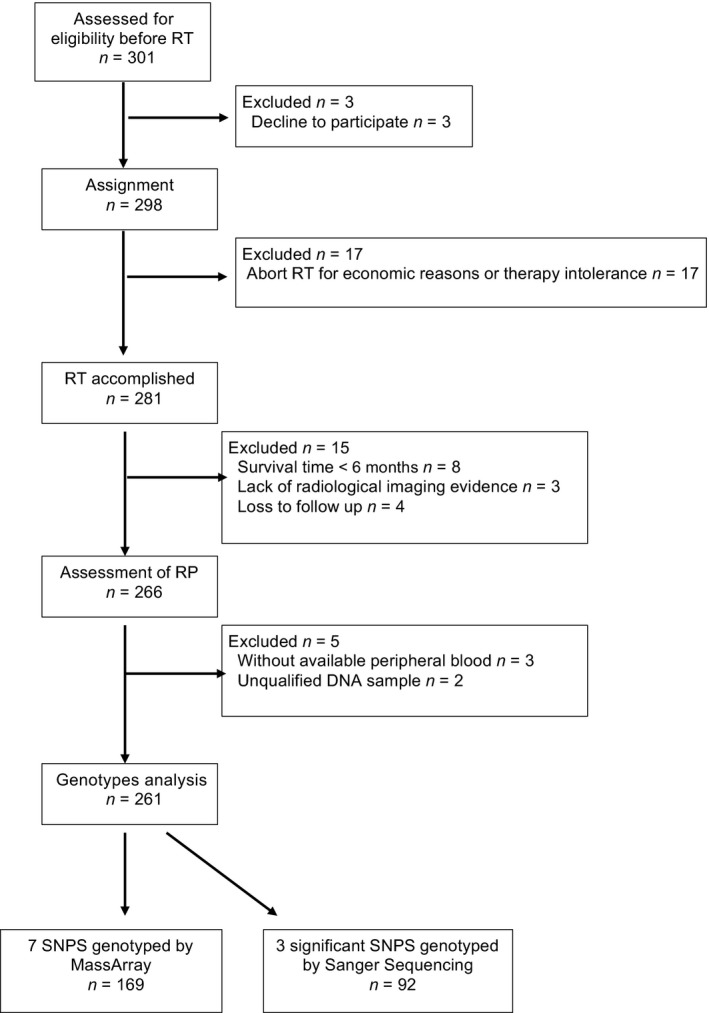
Patient flow diagram.

For our prospective study (NCT02490319), 301 lung cancer patients were initially enrolled. All patients were treated with radiation therapy at Tongji Hospital, Huazhong University of Science and Technology (Wuhan, Hubei Province, China) between 2008 and 2014. We included the patients with a radiation dose of at least 45 Gy, age > 18 years old, KPS > 60, and a life expectancy of at least 6 months. Patients with previous thoracic irradiation or severe cardiopulmonary diseases were excluded from our study. Of the 301 patients, 261 patients (198 with non‐small cell lung cancer and 63 with small‐cell lung cancer) were eventually included for the final genotyping analysis (Fig. 1). Samples from 169 patients were firstly used to genotype the seven candidate SNPs by MassArray to screen for the RP susceptibility variants. And the significant SNPs were then genotyped by Sanger Sequencing in the remaining 92 patients. This study was approved by the Review Board of Tongji Hospital. Written informed consents were obtained from all patients for the use of their clinical information and for obtaining their blood and DNA.

### Treatment and follow‐up

All patients received radiotherapy with 6‐MV X‐rays from a linear accelerator (Elekta Synergy, Elekta, Sweden). The median total radiation dose was 54 Gy (range: 45 to 66 Gy), with 1.5 to 2 Gy administered per radiation treatment. IMRT (intensity‐modulated radiation therapy) was administered to 62.1% of patients (*n* = 162). Computed tomography simulation (CT/e, GE, Fairfield, CT) was performed before the RT treatment was planned. The target volumes and critical normal organs were delineated by the three‐dimensional planning system (Pinnacle Version 9.2, Andover, MA, USA). The baseline clinical characteristics and treatment details of the patients are shown in Table 1.

**Table 1:** Patient characteristics and association between Patient‐, Tumor‐, and Therapy‐Related Characteristics and Grade ≥ 3 Radiation Pneumonitis (*n* = 261)

Parameter	No. of patients	Univariate analysis			Multivariate analysis		
		HR	95% CI	*P*	HR	95% CI	*P*
Gender							
Male	198	1			1		
Female	63	0.581	0.243–1.394	0.224	0.679	0.217–2.126	0.507
Age, years							
<58	121	1			1		
≥58	140	2.121	1.048–4.292	0.037	2.185	1.021–4.678	0.044
Histology							
SCLC	87	1			1		
NSCLC	174	1.062	0.534–2.114	0.864	2.107	0.922–4.816	0.077
Squamous cell	77	0.857	0.361–2.033	0.726	1.057	0.407–2.735	0.911
Adenocarcinoma	79	1.132	0.509–2.521	0.761	2.406	0.939–6.163	0.067
Others	18	1.641	0.529–5.090	0.391	1.946	0.562–6.739	0.294
Stage							
I–II	26	1			1		
III–IV	235	0.700	0.273–1.797	0.459	0.703	0.250–1.981	0.505
KPS							
80–100	197	1			1		
<80	64	1.158	0.561–2.393	0.691	1.119	0.518–2.418	0.775
Smoking							
Nonsmoker	159	1			1		
Smoker	102	1.586	0.784–3.210	0.200	1.116	0.429–2.903	0.822
Surgery							
No	136	1			1		
Yes	125	0.600	0.311–1.156	0.127	0.611	0.268–1.394	0.242
Induction chemotherapy							
No	10	1	1		1		
Yes	251	0.635	0.153–2.642	0.533	0.689	0.151–3.149	0.631
CRT							
No	192	1			1		
Yes	69	1.560	0.794–3.064	0.197	1.849	0.882–3.874	0.104
IMRT							
No	162	1			1		
Yes	99	0.560	0.294–1.067	0.078	0.456	0.226–0.920	0.028
Radiation dose, cGy							
<5400	115	1			1		
≥5400	146	1.940	0.958–3.925	0.066	1.381	0.641–2.978	0.410
aMLD, cGy							
<1500	190	1			1		
≥1500	71	2.783	1.460–5.304	0.002	2.929	1.397–6.140	0.004
aV_20_							
<24%	120	1			1		
≥24%	141	2.393	1.158–4.945	0.018	2.823	1.271–6.267	0.011
COPD							
No	23	1			1		
Yes	238	1.274	0.451–3.597	0.647	1.141	0.379–3.437	0.822

Multivariate analyses were adjusted for all of the factors in this table. KPS, Kamofsky performance status; CRT, concurrent chemoradiation; IMRT, intensity‐modulated radiation therapy; MLD, mean lung dose; V_20_, volume of normal lung receiving 20 Gy or more radiation; COPD, chronic obstructive pulmonary disease.

a Either MLD or V_20_ was used in the multivariate analyses, but not both.

All patients enrolled in this study were examined during and 1 month after radiotherapy. Then, the patients were followed every 3 months for the first year and every 6 months thereafter. At each follow‐up visits, all patients were asked to undergo a chest X‐ray or CT and clinical information, including symptoms, was collected. RP was graded by two radiation oncologists according to the Common Terminology Criteria for Adverse Events 4.0 as follows: Grade 0, no change; Grade 1, asymptomatic and diagnosed by radiographic findings only; Grade 2, symptomatic, not interfering with daily activities; Grade 3, symptomatic, interfering with daily activities or oxygen required; Grade 4, assisted ventilation required; Grade 5, fatal.

### Genotyping methods

We collected the patients' blood 1 month before the initiation of the radiation therapy. Genomic DNA was extracted with a PureLink Genomic DNA Mini Kit (Invitrogen, K1820‐01 Invitrogen, Waltham, MA, USA) from peripheral blood. Haploview software was used to choose the SNPs in PI3K/AKT pathway. We chose three core genes (*PI3CA, AKT1, AKT2*) along PI3K/AKT pathway by the candidate gene approach 23. All of the SNPs had minor allele frequencies >5% in the Chinese population based on the HapMap HCB data, and all correlated alleles were captured at *r* ^2^ > 0.8. And we only selected the SNPs that are located at 5′ or 3′‐UTR gene region, predicted to be functional by bioinformatics analysis and were found to be associated with cancer by previous reports. Seven single‐nucleotide polymorphisms (SNPs) were selected eventually (Table 2). For all seven SNPs, genotypes were firstly determined using the MassArray system (Sequenom iPLEX assay, San Diego, CA). The sample DNA was amplified by a multiplex PCR reaction, and the PCR products were then used for a locus‐specific single‐base extension reaction. Finally, the resulting products were desalted and transferred to a 384‐element SpectroCHIP array. The alleles were discriminated by mass spectrometry (Sequenom). After analysis, three RP susceptibility SNPs, *PI3CA*: rs9838117, *AKT2*: rs33933140, and *AKT2*: rs11880261 were then genotyped by Sanger Sequencing method in the remaining 92 patients. The primer pairs for rs9838117 were F: 5′‐CTCAGCAGGCAAAGACCG‐3′; R: 5′‐ CAAGAGTCCCTTCCACCC‐3′, for rs33933140 were F: 5′‐CCCCAAATGTTCCTCCTGC‐3′; R: 5′‐ACGCTCGCTGCCATCAC‐3′, and for rs11880261 were F: 5′‐AGAAATGCTGGACTGGCTGTAGG‐3′; R: 5′‐CAGAGGTCTCATAGCTGAAGTGGG‐3′. The PCR products were then subjected to DNA sequencing to detect mutations.

**Table 2:** Genes and single‐nucleotide polymorphisms selected for analysis

Genes and single‐nucleotide polymorphism	Allelic change	Functional consequence
PIK3CA		
rs2699887	A/G	Upstream variant 2KB
rs6443626	C/T	Utr variant 3 prime
rs9838117	G/T	Utr variant 3 prime
AKT1		
rs2498786	C/G	Upstream variant 2KB
AKT2		
rs11880261	C/T	Utr variant 5 prime
rs33933140	A/G	Utr variant 3 prime
rs7254617	A/G	Upstream variant 2KB

### Statistical analysis

The end point for this study was the development of RP ≥ grade 3. The time to the end point was calculated from the start of radiotherapy. Patients who did not experience RP ≥ grade 3 within 12 months of RT were censored. The definition of smoker is the person who has or had smoked for more than 6 months, including former smoker and current smoker. And the nonsmoker means the person who has never smoked or has/had smoked for <6 months. SPSS 21.0 statistical software (SPSS, lnc., Chicago, IL) was used for the statistical analysis. Patients were divided into groups according to their genotypes, and Cox proportional hazard analysis was applied to estimate the hazard ratio (HR) and 95% confidence intervals (CIs) of all factors possibly related to the risk of RP. Moreover, multivariate Cox regression analysis was used for the adjustment of covariates. The influences of the genotypes on RP risk were assessed by Kaplan–Meier analysis and compared with log‐rank tests. For genotype analysis, *P*‐values were corrected by the Benjamini and Hochberg False Discovery Rate correction.

## Results

### Patient characteristics and radiation pneumonitis

Two hundred and sixty one patients were included in this study with 198 males and 63 females. Their characteristics are listed in Table 1. The median age of the population was 58 years (range from 29 to 79 years); 174 patients had NSCLC, and 87 had SCLC. In the study cohort, 90% of patients had stage III–IV disease, 52.1% underwent surgery before RT, almost all patients (96.2%) received induction chemotherapy before radiotherapy, 26.4% had concurrent chemoradiation, and 25.3% patients received both induction chemotherapy and concurrent chemoradiation. The median radiation dose was 54 Gy (range from 45 to 66 Gy), the median MLD was 13.50 Gy (range from 1.78 to 20.17 Gy), and the median V_20_ was 24.15% (range from 0 to 42.00%). We used 15 Gy as the cutoff value of MLD in our cohort because MLD ≥ 15 Gy is an important domestic predictor of severe RP occurrence and has been used by many published researches 14, 24. The characteristics of 169 patients, who were firstly genotyped by MassArray to screen for RP susceptibility variants, are listed in Table S1.

Within 12 months of radiotherapy, 37 patients (14.2%) suffered RP ≥ grade 3. The associations between patient‐, tumor‐, and therapy‐related characteristics and RP ≥ grade 3 are listed in Tables 1. The univariate and multivariate analysis by Cox regression model revealed that MLD, V_20_, age, IMRT was significantly related to RP ≥ grade 3. Patients with elder age, MLD ≥ 15 Gy, V_20_ ≥ 24% had higher risk of RP ≥ grade 3 compared with those counterparts (HR = 2.185, 95% CI: 1.021–4.678, *P *=* *0.044; HR = 2.929, 95% CI: 1.379–6.140, *P *=* *0.004; HR = 2.823, 95% CI: 1.271–6.267, *P *=* *0.011, respectively). Moreover, patients receiving IMRT were associated with decreased incidence of RP (HR = 0.456, 95% CI: 0.226–0.920, *P *=* *0.028) (Table 1), which were consistent with the results of other publications 5.

### *PI3CA* and *AKT2* SNPs and RP

**Figure 2:**
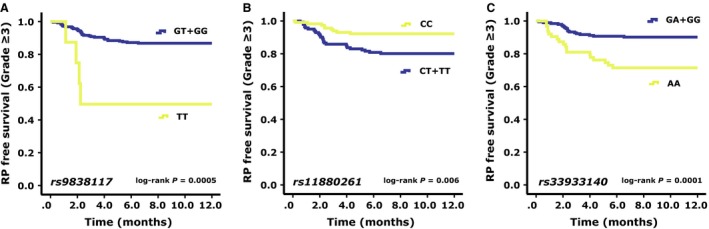
Kaplan–Meier estimates RP‐free survival (RP ≥ grade 3) for each genotype. (A) *PI3CA*: rs9838117; (B) *AKT2*: rs11880261 (C) *AKT2*: rs33933140. Patients with GT/GG genotype of *PI3CA*: rs9838117 (*P *=* *0.0005), the CC genotype of *AKT2*: rs11880261 (*P *=* *0.006), and the GA/GG genotype of *AKT2*: rs33933140 (*P *=* *0.0001) had significantly lower risks of RP ≥ grade 3.

Seven candidate SNPs in PI3K/AKT pathway were screened by MassArray in 169 patients for RP susceptibility variants (Table 2). Among them, *PI3CA*: rs9838117, *AKT2*: rs33933140, and *AKT2*: rs11880261 were found to be significantly associated with the occurrence of RP ≥ grade 3 (Table S3). The above three SNPs were then genotyped by Sanger Sequencing method in the remaining 92 patients, bringing the total population of 261 patients genotyped for these three SNPs. Figure 2 is a plot of the RP‐free survival percentage for RP ≥ grade 3 for each genotype of *PI3CA*: rs9838117, *AKT2*: rs33933140, and *AKT2*: rs11880261 determined by the Kaplan–Meier method. Patients with the GT/GG genotype of *PI3CA*: rs9838117 (*P *=* *0.0005, Fig. 2A), the CC genotype of *AKT2*: rs11880261 (*P *=* *0.006, Fig. 2B), and the GA/GG genotype of *AKT2*: rs33933140 (*P *=* *0.0001, Fig. 2C) had significantly lower risks of RP ≥ grade 3. Furthermore, multiple Cox proportional hazard analyses with adjustments for all of the characteristics listed in Table 1 revealed that the GA/GG genotype of *AKT2*: rs33933140 (HR = 0.272, 95% CI: 0.140–0.530, *P *=* *1.3E–4, *P*_*c*_ = 9.1E–4), and the CG/GG genotype of *PI3CA*: rs9838117 (HR = 0.132, 95% CI: 0.042–0.416, *P *=* *0.001, *P*_*c*_ = 0.006) were strongly related to a decreased occurrence of RP ≥ grade 3. Moreover, patients with the CT/TT genotype of *AKT2*: rs11880261 had a notably higher incidence of RP ≥ grade 3 (HR = 2.950, 95% CI: 1.380–6.305, *P *=* *0.005, *P*_*c*_ = 0.025) (Tables 3).

**Table 3:** Association between genotypes and Grade ≥ 3 RP

Polymorphism and Genotype	No. of event	No. of total	Univariate analysis			Multivariate analysis			P_c_
			HR	95% Cl	*P*	HR	95% Cl	*P*	
*AKT2*: rs33933140									
AA	18	64	1			1			
GA+GG	19	197	0.309	0.162–0.589	3.6E–4	0.272	0.140–0.530	1.3E–4	9.1E–4
*AKT2*: rs11880261									
CC	9	117	1			1			
CT + TT	28	144	2.726	1.286–5.778	0.009	2.950	1.380–6.305	0.005	0.025
*PI3CA*: rs9838117									
TT	4	8	1			1			
GT + GG	33	253	0.191	0.067–0.540	0.002	0.132	0.042–0.416	0.001	0.006

Multiple analyses in this table were adjusted for all of the factors listed in Table 1. HR, hazard ratio; CI, confidence interval.

*P*_*c*_: *P*‐value corrected by Benjamini and Hochberg False Discovery Rate correction.

### *AKT2*: rs33933140 and dosimetric factors

**Figure 3:**
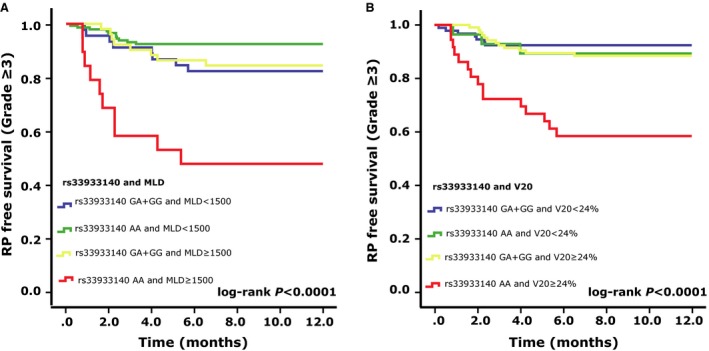
Kaplan–Meier estimates effect of genotype in rs33933140 and dosimetric parameters on RP‐free survival (RP ≥ grade 3). (A) rs33933140 and MLD; (B) rs33933140 and V_20_. Patients with AA genotype of *AKT2*: rs33933140 and MLD ≥ 15 Gy or V_20_ ≥ 24% had the highest risk of RP grade ≥ 3 compared with other groups (*P *<* *0.0001 and *P *<* *0.0001, respectively).

Patients were divided to four groups based on the dosimetric factors‐V_20_ or MLD and rs33933140 genotypes in order to evaluate the impact of the rs33933140 genotypes on RP in different dosimetric groups. Patients with AA genotype of *AKT2*: rs33933140 and MLD ≥ 15 Gy or V_20_ ≥ 24% had the highest risk of RP grade ≥ 3 compared with other groups (*P *<* *0.0001 and *P *<* *0.0001, respectively, Fig. 3A, B). Interestingly, for the patients with *AKT2*: rs33933140 GA/GG genotype and MLD ≥ 15 Gy or V_20_ ≥ 24%, they had the same incidence of RP ≥ grade 3 with the patients who received MLD <15 Gy or V_20_ < 24%, suggesting the dominant and independent role of rs33933140 genotypes in severe RP.

## Discussion

Current study evaluated the genetic variants of *PIK3CA, AKT1, and AKT2,* all of which are genes in PI3K/AKT pathway, to discover their potential associations with the occurrence of RP in lung cancer patients with radiotherapy. Among them, three SNPs, *PI3CA*: rs9838117, *AKT2*: rs33933140, and *AKT2*: rs11880261, were found to be significantly associated with the occurrence of RP ≥ grade 3. Patients with the TT genotype of *PI3CA*: rs9838117, the AA genotype of *AKT2*: rs33933140, and the CT+TT genotype of *AKT2*: rs11880261 had a significantly increased risk of RP after radiotherapy for lung cancer. We also discovered that the association between rs33933140 and RP grade ≥ 3 was independent of MLD and V_20_, and further identified a group of patients with the highest risk of severe RP (patients with AA genotype of rs33933140 and MLD ≥ 15 Gy or V_20_ ≥ 24%). To the best of our knowledge, it is the first study to address the associations between genetic variants in PI3K/AKT pathway and RP risk.

The occurrences of RP ≥ grade 3 were 14.2%, which were similar to those reported previously 14. Due to the prospective nature of our study, the incidence rate of RP was relatively higher than in some retrospective studies 6. We also confirmed that age, MLD, and V_20_ was closely related to the risk of RP. In our cohort, patients with age ≥ 58, MLD ≥ 15 Gy, and V_20_ ≥ 24%, and without receiving IMRT had a greater risk of developing RP grade ≥ 3, which verified the associations between the patient‐ and radiation dosimetric‐related factors and the occurrence of severe RP.

As a complication after the thoracic irradiation, RP is characterized by inflammation and subsequent fibrosis of the lung. It is well‐known that proinflammatory and fibrogenic cells and cytokines induced by irradiation are involved in the pathogenesis of RP. However, the molecular mechanisms of RP pathogenesis are poorly defined and remain under intensive research. Genetic variants of *PI3CA*, which is the catalytic domain for PI3K and *AKT2* were found to be notably related to severe RP in our cohort. The PI3Ks are intracellular lipid kinases and can catalyze the phosphorylation of phosphatidylinositol 3,4‐bisphosphate (PIP2) to phosphatidylinositol 3,4,5‐triphosphate (PIP3), allowing the phosphorylation of AKT 25, 26, 27. The PI3K/AKT pathway has been proved to be involved in multiple cellular processes including cell proliferation, apoptosis, survival, inflammation, and fibrosis. It is well‐known that the PI3K/AKT pathway can regulate several determinant events in the inflammatory response to injury. Several studies demonstrated that the PI3K inhibitors or genetic loss of PI3K causes reduction in recruitment and activation of lymphocytes, neutrophils, macrophages, and eosinophils in a variety of in vitro and in vivo researches. Moreover, PI3Ks play important roles in T and B cell development, survival, proliferation, and differentiation 28, 29. Activated AKT, composed of three major isoforms AKT1, 2, 3, can also regulate FOXO transcription factors, which are crucial regulators of immune homeostasis^18^. Meanwhile, PI3K/AKT pathway also plays important roles in pulmonary fibrosis. Upexpression of PI3K in myofibroblasts and bronchiolar basal cells was found in idiopathic pulmonary fibrosis 30. In the pathogenesis of RP, lung epithelial cells are demonstrated to transdifferentiate into fibroblast‐like cells by epithelial‐to‐menschymal transition (EMT). PI3K/AKT signaling pathway is a critical mediator of EMT. Moreover, PI3K/AKT can suppress FoxO3a, which is a critical promoter of the apoptosis in fibroblasts 31. PI3K/AKT can also affect adhesion of fibroblasts with fibronectin and collagen leading to deposition of extracellular matrix by interaction with integrin pathways 19. Moreover, inhibition of PI3K was proved to prevent the proliferation and differentiation of human lung fibroblasts into myofibroblasts in vitro and the PI3K inhibitors, WO2013117503 and WO2013117504 have been used to treat idiopathic pulmonary fibrosis in Phase I study 32, 33. Generally speaking, the above evidence suggested that the PI3K/AKT pathway is a vital regulator in inflammation and fibrosis, indicating the biological plausibility of the relevance of PI3K/AKT to RP found in our research.

Our study suggested in addition to the radiation dosimetric factors that SNPs in *PI3CA*: rs9838117, *AKT2*: rs33933140, and *AKT2*: rs11880261 genotyping assays can be used as predictive biomarkers of RP risk before RT. Thus, patients with these RP susceptibility genotypes will greatly benefit from early prediction and prevention of RP by genotyping before the initiation of RT. And this study will help us to choose the patients without RP susceptibility genotypes and elevate their radiation dose appropriately for a better control of tumor. Especially for the patients with favorable genotypes, elevated MLD and V_20_ will not increase their incidence of severe RP, which could assist the oncologist to adjust the radiation dose personally. Moreover, our findings suggest the possible role of PI3K/AKT pathway in the pathogenesis of RP, which will aid in the discovery of target to treat RP in future research.

However, because of the relatively small population in this study, our findings need to be confirmed by larger, multicentered studies. Due to the substantial ethnic variation in SNP frequencies, our results, which were demonstrated in a Han Chinese population, should be validated in different races. Moreover, rs9838117, rs33933140, and rs11880261 warrant further investigation to identify the causative SNPs and their molecular mechanisms. To this end, a luciferase reporter vector (pGL3‐basic) containing the rs33933140 SNP in the *AKT2* 3′‐UTR region, is currently being developed to discover the molecular impact of the rs33933140 SNP on AKT2 expression. Furthermore, we need to explore the potential role of PI3K/AKT pathway in the pathogenesis of RP, which would provide novel insight into the treatment of RP. On the other hand, it is well‐known that the PI3K/AKT pathway is one of the most important signaling pathways in cancer progression. So we performed statistical analysis on the association between these three SNPs and TTP (Time To Progression) after radiotherapy. However, we did not find significant relations between them by Cox regression analysis (Table S4). In the future study, we will do more research on discovering the impact of SNPs in PI3K/AKT pathway on the cancer progression in our cohort.

In conclusion, this is the first study to evaluate the associations between genetic variations in the PI3K/AKT pathway and RP. Three SNPs in PI3K/AKT pathway, *PI3CA*: rs9838117, *AKT2*: rs33933140, and *AKT2*: rs11880261 were found to be significantly associated with the occurrence of RP ≥ grade 3 and may thus serve as predictors of RP if it is further validated in larger population. Our study also provides support for the further research about the roles of PI3K/AKT pathway in the pathogenesis of RP.

## Conflict of Interest

The authors have no conflict of interest.

## Supporting information


**Table S1.** Patient Characteristics (*N* = 169).
**Table S2.** Association between Patient‐, Tumor‐, and Therapy‐Related Characteristics and Grade ≥ 3 Radiation Pneumonitis (*N* = 169).
**Table S3.** Association between genotypes and Grade ≥ 3 RP (*N* = 169).
**Table S4.** Association between genotypes and TTP (*N* = 261).
