# Supplementary material for: Genetic variants in PI3K/AKT pathway are associated with severe radiation pneumonitis in lung cancer patients treated with radiation therapy
Source: Cancer Med. 2015 Dec 8;5(1):24–32. doi: 10.1002/cam4.564 (PMC4708901; doi:10.1002/cam4.564)
Supplement: Supplementary file 1 — Table S1. Patient Characteristics (N = 169). Table S2. Association between Patient‐, Tumor‐, and Therapy‐Related Characteristics and Grade ≥ 3 Radiation Pneumonitis (N = 169). Table S3. Association between genotypes and Grade ≥ 3 RP (N = 169). Table S4. Association between genotypes and TTP (N = 261). [file CAM4-5-024-s001.doc]

| **S. Table 1. Patient Characteristics (N=169)** | | | |
| --- | --- | --- | --- |
| **Characteristic** | | **No. of Patients** | **%** |
| **Sex** | Male  Female | 125  44 | 74.0  26.0 |
| **Age, years** | Median  Range | 57  28-78 |  |
| **Histology** | SCLC  NSCLC | 55  114 | 32.5  67.5 |
| **Stage** | I- II  III-IV | 24  145 | 10.2  85.8 |
| **KPS** | 80-100  <80 | 123  46 | 72.6  27.4 |
| **Smoking** | Smoker  Non-smoker | 106  63 | 62.0  38.0 |
| **Chemotherapy** | Yes  No | 160  9 | 94.7  5.3 |
| **CRT** | Yes  No | 44  125 | 26.0  74.0 |
| **Surgery** | Yes  No | 86  83 | 50.9  49.1 |
| **IMRT** | Yes  No | 79  90 | 46.7  53.3 |
| **Radiation dose**  **(cGy)** | Median  Range | 5600  4500-6600 |  |
| **MLD**  **(cGy)** | Median  Range | 1368  178-2017 |  |
| **V_20_** | Median  Range | 24.82  0-42.00 |  |
| **COPD** | Yes  No | 19  150 | 11.2  88.8 |

Abbreviations: KPS, Kamofsky performance status; CRT, concurrent chemoradiation; IMRT, intensity-modulated radiation therapy; MLD, mean lung dose; V_20_, volume of normal lung receiving 20 Gy or more radiation; COPD, chronic obstructive pulmonary disease.

| **S.Table2. Association between Patient-, Tumor-, and Therapy-Related Characteristics and Grade≥3 Radiation Pneumonitis (N=169)** | | | | | | | | | |
| --- | --- | --- | --- | --- | --- | --- | --- | --- | --- |
| **Parameter** |  | | **Univariate Analysis** | | | | **Multivariate Analysis** | | |
|  |  | **HR** | | **95%CI** | **P** | **HR** | | **95%CI** | **P** |
| **Sex** | Female  Male | 1  1.604 | | 0.660-3.897 | 0.297 | 1  1.455 | | 0.442-4.791 | 0.537 |
| **Age, years** | <57  ≥57 | 1  1.838 | | 0.886-3.813 | 0.102 | 1  2.139 | | 0.975-4.693 | 0.058 |
| **Histology** | SCLC  NSCLC | 1  1.071 | | 0.507-2.261 | 0.858 | 1  1.916 | | 0.758-4.840 | 0.169 |
| **Stage** | I-II  III-IV | 1  0.877 | | 0.308-2.501 | 0.806 | 1  0.796 | | 0.242-2.616 | 0.707 |
| **KPS** | 80-100  <80 | 1  1.66 | | 0.493-2.305 | 0.870 | 1  1.001 | | 0.431-2.322 | 0.999 |
| **Smoking** | Smoker  Nonsmoker | 1  0.670 | | 0.317-1.414 | 0.293 | 1  0.964 | | 0.337-2.755 | 0.945 |
| **Surgery** | Yes  No | 1  1.383 | | 0.688-2.781 | 0.363 | 1  0.775 | | 0.316-1.900 | 0.577 |
| **Chemotherapy** | Yes  No | 1  0.947 | | 1  0.129-6.935 | 0.957 | 1  0.885 | | 0.103-7.616 | 0.911 |
| **CRT** | Yes  No | 1  0.648 | | 0.312-1.344 | 0.244 | 1  0.607 | | 0.275-1.339 | 0.216 |
| **IMRT** | Yes  No | 1  1.029 | | 0.514-2.059 | 0.937 | 1  1.179 | | 0.773-1.698 | 0.498 |
| **Radiation dose, cGy** | ＜5600  ≥5600 | 1  1.294 | | 0.639-2.621 | 0.473 | 1  1.139 | | 0.533-2.435 | 0.737 |
| **MLD，cGy** | <1500  ≥1500 | 1  2.353 | | 1.175-4.714 | 0.016 | 1  2.672 | | 1.225-5.827 | 0.014 |
| **V20** | <24%  ≥24% | 1  2.334 | | 1.049-5.197 | 0.038 | 1  2.966 | | 1.231-7.151 | 0.015 |
| **COPD** | Yes  No | 1  0.639 | | 0.246-1.661 | 0.358 | 1  0.680 | | 0.238-1.938 | 0.470 |

Note: Multivariate analyses were adjusted for all factors in S.Table1.

Abbreviations: HR, hazard ratio; KPS, Kamofsky performance status; RT, radiotherapy; CRT, concurrent chemoradiation; IMRT, intensity-modulated radiation therapy; MLD, mean lung dose; V_20_, volume of normal lung receiving 20Gy or more radiation; COPD, chronic obstructive pulmonary disease.

*Either MLD or V_20_ was used in multivariate analyses, but not together.

|  | | | | | | | | | | | | | | | | | | |  | |
| --- | --- | --- | --- | --- | --- | --- | --- | --- | --- | --- | --- | --- | --- | --- | --- | --- | --- | --- | --- | --- |
| **S.Table3**. Association between genotypes and Grade ≥3 RP (N=169) | | | | | | | | | | | | | | | | | | | | |
| Polymorphism and Genotype | | No.of event | | | No.of total | | Univariate analysis | | | | | | Multivariate analysis | | | | | |  | |
|  |  |  |  |  |  |  | HR | | 95% CL | | P | | HR | | 95% CL | | P | | P_c_ | |
| *PI3CA*:rs6443626 | |  | | |  | |  | |  | |  | |  | |  | |  | |  | |
|  | TT  CT+CC | 23  10 | | | 138  31 | | 1  2.028 | | 0.965-4.263 | | 0.062 | | 1  1.314 | | 0.859-2.010 | | 0.208 | | 0.832 | |
| *PI3CA*:rs9838117 | |  | | |  | |  | |  | |  | |  | |  | |  | |  | |
|  | TT  GT+GG | 3  30 | | | 6  163 | | 1  0.277 | | 0.084-0.911 | | 0.035 | | 1  0.212 | | 0.063-0.712 | | 0.012 | | 0.06 | |
| *PI3CA*:rs2699887 | |  | | |  | |  | |  | |  | |  | |  | |  | |  | |
|  | CC  CT | 32  1 | | | 152  17 | | 1  0.244 | | 0.033-1.788 | | 0.165 | | 1  0.287 | | 0.038-2.194 | | 0.229 | | 0.687 | |
| *AKT1:*rs2498786 | | | | |  | |  | |  | |  | |  | |  | |  | |  | |
|  | CC  CG+GG | 18  15 | | | 107  62 | | 1  0.692 | | 0.349-1.373 | | 0.292 | | 1  0.804 | | 0.363-1.779 | | 0.590 | | 1.180 | |
| *AKT2:*rs7254617 | |  | | |  | |  | |  | |  | |  | |  | |  | |  | |
|  | GG  GA+AA | 22  11 | | | 152  47 | | 1  1.230 | | 0.596-2.537 | | 0.575 | | 1  1.066 | | 0.504-2.255 | | 0.866 | | 0.866 | |
| *AKT2*:rs33933140 | |  | | |  | |  | |  | |  | |  | |  | |  | |  | |
|  | AA  GA+GG | 15  18 | | | 41  128 | | 1  0.341 | | 0.172-0.676 | | 0.002 | | 1  0.338 | | 0.170-0.672 | | 0.002 | | 0.014 | |
| *AKT2*: rs11880261 | | |  |  | |  | |  | |  | |  | |  | |  | |  | |  |
|  | CC  CT+TT | 8  25 | | | 77  92 | | 1  2.938 | | 1.325-6.514 | | 0.008 | | 1  3.363 | | 1.473-7.678 | | 0.004 | | 0.024 | |

NOTE: Multiple analyses in this table were adjusted for all the factors listed in S.Table 1.

Abbreviations: HR, hazard ratio；CI, confidence interval.

P_c_: P-value corrected by Benjamini and Hochberg False Discovery Rate correction.

**S.Table4**. Association between genotypes and TTP (N=261)

| Polymorphism and Genotype | | | No.of total | | Univariate analysis | | | | | | Multivariate analysis | | | |
| --- | --- | --- | --- | --- | --- | --- | --- | --- | --- | --- | --- | --- | --- | --- |
|  |  |  |  |  | HR | | 95% CL | | P | | HR | | 95% CL | P |
| *AKT2*:rs33933140 | | |  | |  | |  | |  | |  | |  |  |
|  | AA  GA+GG | | 64  197 | | 1  0.873 | | 0.617-1.236 | | 0.445 | | 1  0.926 | | 0.645-1.328 | 0.676 |
| *AKT2*: rs11880261 | | | |  | |  | |  | |  | |  | | |
|  | CC  CT+TT | | 117  144 | | 1  0.926 | | 0.684-1.254 | | 0.620 | | 1  0.873 | | 0.638-1.194 | 0.395 |
| *PI3CA*:rs9838117 | | |  | |  | |  | |  | |  | |  |  |
|  | | TT  GT+GG | 8  253 | | 1  0.495 | | 0.232-1.055 | | 0.069 | | 1  0.487 | | 0.223-1.064 | 0.071 |

NOTE: Multiple analyses in this table were adjusted for age, sex, smoking state, stage, histology, KPS, radiation dose, chemotherapy and IMRT.

Abbreviations: TTP, time to progression; HR, hazard ratio；CI, confidence interval.
